# Colorectal adenoma recurrence rates among post-polypectomy patients in the placebo-controlled groups of randomized clinical trials: a meta-analysis

**DOI:** 10.18632/oncotarget.18181

**Published:** 2017-05-25

**Authors:** Xin Shi, Zhiping Yang, Qiong Wu, Daiming Fan

**Affiliations:** ^1^ State Key Laboratory of Cancer Biology, National Clinical Research Center for Digestive Diseases and Xijing Hospital of Digestive Diseases, Fourth Military Medical University, Xi’an, China

**Keywords:** colorectal adenoma, placebo, recurrence, meta-analysis

## Abstract

**Background:**

Evidence regarding the benefit of therapy to prevent the post-polypectomy recurrence of colorectal adenoma is limited. Endoscopic recurrence is the main outcome according to an evaluation of trials involving recurrence prevention.

**Aim:**

To estimate the recurrence rates of post-polypectomy colorectal adenoma in placebo-controlled arms of randomized clinical trials and to identify the prognostic factors influencing these rates.

**Methods:**

We combined data from all randomized controlled trials evaluating therapies for colorectal adenoma using placebo from 1988 to 2016. The data were combined in a random-effects model. Primary outcomes were endoscopic adenoma and advanced adenoma recurrence of colorectal adenoma.

**Results:**

The pooled estimates of the adenoma recurrence rates were 37% (95% confidence interval [CI], 33%-41%; range, 33%-52%) at 1 year, 47% (95% CI, 41%-54%; range, 46%-51%) at 2 years, 41% (95% CI, 33%-48%; range, 20%-61%) at 3 years, 48% (95% CI, 38%-57%; range, 37%-53%) at 4 years, and 60% (95% CI, 52%-68%; range, 48%-68%) at 5 years. The pooled estimates of the advanced adenoma recurrence rates were 10% (95% CI, 6%-15%; range, 7%-13%) at 1 year, 12% (95% CI, 8%-16%; range, 3%-19%) at 3 years, 14% (95% CI, 10%-18%; range, 13%-16%) at 4 years, and 14% (95% CI, 10%-19%; range, 9%-21%) at 5 years. Significant heterogeneity among the randomized clinical trials (P < 0.001) was observed for each recurrence rate.

**Conclusions:**

This meta-analysis confirms the heterogeneity of recurrence rates among post-polypectomy colorectal adenoma patients who received placebo. No single design variable was identified that might explain the heterogeneity.

## INTRODUCTION

As a malignant neoplasm, colorectal cancer is common worldwide, and its prevalence and case fatality are increasing [1]. Occurring within the lining of the large intestine, approximately 85% of colorectal cancers are believed to develop from adenomatous polyps, a process termed the adenoma-to-carcinoma sequence [2]. Although colorectal adenoma resection interrupts the progression to invasive disease [3], patients with adenoma (treated by polypectomy) remain at high risk for colorectal adenoma recurrence or the development of colorectal cancer [4]. Thus, a transition from surveillance for the early detection of cancer and adenoma to new approaches for prevention such as chemoprevention is required to relieve the burden of this disease.

Several large multicentre randomized double-blinded placebo-controlled trials have assessed the possible preventive effect of various agents on the recurrence of colorectal adenoma after polypectomy, including antioxidant vitamins, calcium, fibre, ursodeoxycholic acid, folic acid, difluoromethylornithine, metformin, and non-steroidal anti-inflammatory drugs, such as aspirin and selective COX-2 inhibitors [5–27]. Among those randomized controlled trials (RCTs) evaluating drugs for the chemoprevention of recurrence, both adenoma and advanced adenoma recurrence after polypectomy were evaluated as main outcomes.

The rate of colorectal adenoma recurrence in a placebo-controlled group, defined as the rate of patients in a placebo-controlled group with recurrence of one or more adenomas detected by endoscopy, and the rate of endoscopic advanced adenoma recurrence, defined as the rate of patients with recurrence of adenomas with any of the characteristics of diameter 10 mm or more, tubulovillous or villous histology, high-grade dysplasia or carcinoma [28] varied among the studies. In the placebo groups, the rates of endoscopic adenoma and advanced adenoma recurrence ranged from 32.5% [11] to 51.6% [6] and from 7.2% [11] to 12.7% [18] at 1 year, respectively; from 20.2% [23] to 60.7% [13] and from 3.2% [5] to 19.0% [20] at 3 years, respectively; and from 48.2% [7] to 68.4% [13] and from 9.2% [7] to 21.3% [9] at 5 years, respectively.

Therefore, an accurate estimate of the recurrence rate among patients treated with placebo is essential to evaluate the natural history of the disease, calculate sample size, assess the effect size of treatment to formulate preventive strategies, and interpret the results of RCTs examining different treatments.

We conducted a meta-analysis to estimate the 1-year, 2-year, 3-year, 4-year, and 5-year recurrence rates in placebo-controlled groups and analysed the variability in recurrence rates by examining the heterogeneity among the studies; we also attempted to interpret this variability.

## RESULTS

### Description of the studies

**Figure 1:**
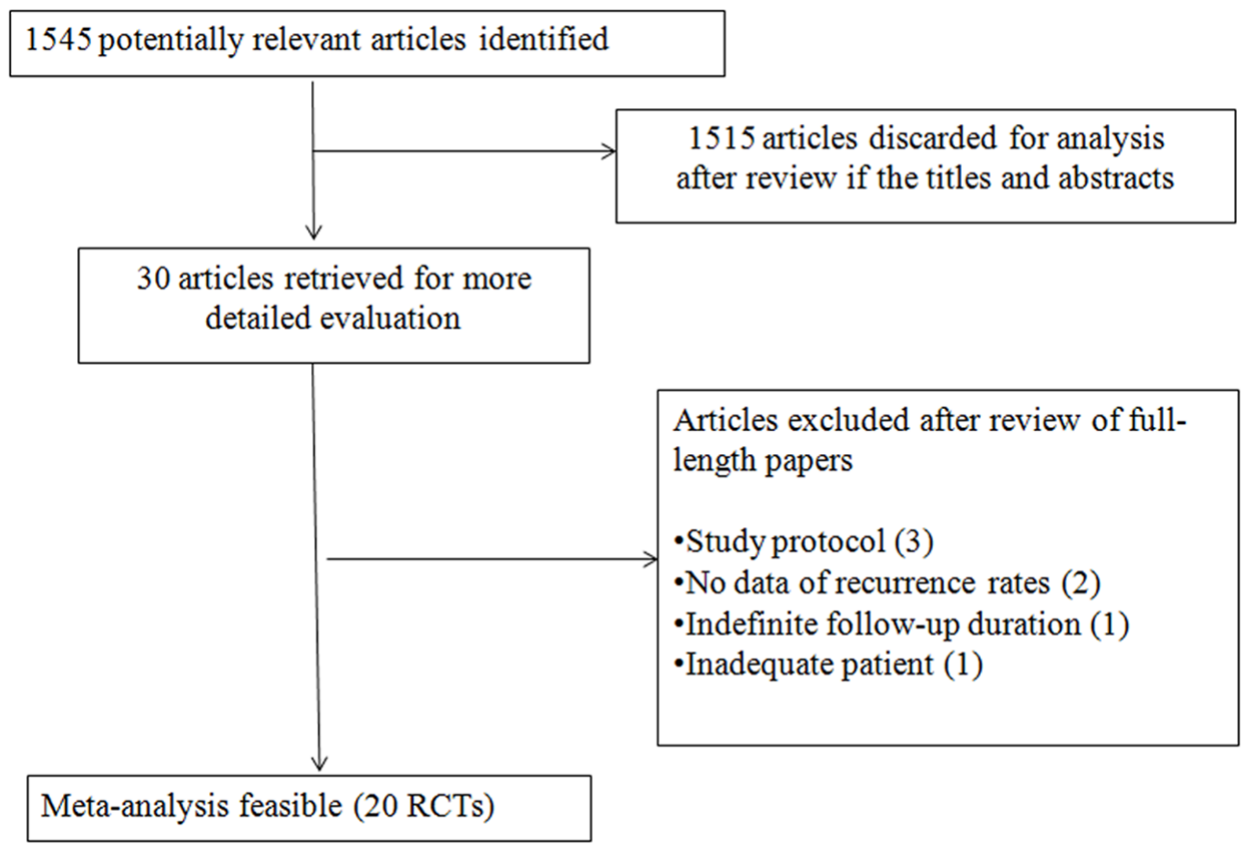
Study flowchart

After reviewing the titles and abstracts, 20 articles [5-16, 18, 20, 22-27] were found to fulfil the inclusion criteria and were selected for review (Figure 1). Four studies [24–27] were published before 2000, and the remaining 16 [5–23] were published after 2000. The distribution of the baseline patient characteristics in the control arm of the 20 studies considered in this meta-analysis is shown in Table 1. The characteristics of the treatment and control arms of the studies considered in our analysis are reported in Supplementary Table 1. All RCTs included in this meta-analysis were double-blind, placebo-controlled trials, except for one [26] in which the control arm received no treatment.

**Table 1:** Study characteristics

First author	Year	Sample size	Centers	Male(%)	Mean age(y)	Aspirin user (%)	Current smoker (%)	Daily calcium (mg/d)	Daily alcohol (g/d)	Daily folate (ug/d)	Family history *(%)	1-year recurrence(%)		2-year recurrence(%)		3-year recurrence(%)		4-year recurrence(%)		5-year recurrence(%)	
												Any	Advanced	Any	Advanced	Any	Advanced	Any	Advanced	Any	Advanced
Mckeown	1988	67	2	70.8	58.4	NA	83.1	NA	NA	NA	NA	NA	NA	50.7	NA	NA	NA	NA	NA	NA	NA
Roncucci	1993	78	2	57.7	60.8	NA	NA	NA	NA	NA	NA	35.9	NA	NA	NA	NA	NA	NA	NA	NA	NA
Greenberg	1994	187	4	82	61	NA	NA	NA	NA	NA	NA	36.4	NA	NA	NA	NA	NA	NA	NA	NA	NA
Baron	1999	423	6	70	61	NA	NA	865	NA	NA	NA	33.0	NA	NA	NA	NA	NA	52.0	NA	NA	NA
Bonithon	2000	178	21	60.1	59.3	NA	NA	1023	NA	NA	15.3	NA	NA	NA	NA	20.2	NA	NA	NA	NA	NA
Baron	2003	363	9	62.6	57.4	35.3	14.3	780	NA	328	28.2	NA	NA	NA	NA	47.1	12.9	NA	NA	NA	NA
Alberts	2005	579	4	66.2	66.4	26.6	12.5	962.1	8.3	NA	29	NA	NA	NA	NA	43.9	19.0	NA	NA	NA	NA
Baron	2006	1202	108	62	59.4	15.7	20.7	NA	NA	NA	21.5	39.2	12.7	NA	NA	54.6	18.0	NA	NA	NA	NA
Cole	2007	486	9	63.6	57	37.8	13.6	NA	8.4	325	37.9	NA	NA	NA	NA	42.4	8.6	NA	NA	65.6	13.0
Logan	2008	204	10	60.9	58	NA	NA	1131.2	12.4	297.6	25.2	NA	NA	NA	NA	27.5	14.7	NA	NA	NA	NA
Meyskens	2008	129	7	75	61	37.5	41.4	NA	NA	NA	NA	NA	NA	NA	NA	41.1	8.5	NA	NA	NA	NA
Bertagnolli	2009	679	91	69.7	59	31.2	18	NA	NA	NA	20.6	NA	NA	NA	NA	60.7	17.2	NA	NA	68.4	21.3
Wu	2009	238	multiple	38	65.7	41	NA	981	9.6	319	32	NA	NA	NA	NA	30.3	7.1	NA	NA	NA	NA
Arber	2011	628	107	64.6	61	17	NA	NA	NA	NA	17.4	32.5	7.2	NA	NA	49.3	10.4	NA	NA	57.5	13.8
Benamouzig	2012	132	49	70	57.7	NA	25.2	NA	NA	NA	39.4	41.1	11.6	NA	NA	NA	NA	53.0	15.5	NA	NA
Bonelli	2013	166	3	65.9	57	NA	NA	NA	NA	NA	NA	NA	NA	NA	NA	NA	NA	37.3	12.6	NA	NA
Ishikawa	2014	159	19	78.6	60.5	NA	21.4	NA	NA	NA	NA	NA	NA	45.9	NA	NA	NA	NA	NA	NA	NA
Baron	2015	380	11	85.5	58.2	38.6	8.4	672	12.5	NA	15.6	NA	NA	NA	NA	NA	NA	NA	NA	48.2	9.2
Higurashi	2016	62	5	79	63.5	NA	40	NA	NA	NA	16	51.6	NA	NA	NA	NA	NA	NA	NA	NA	NA
Pommergaard	2016	218	109	60	60	NA	24.9	NA	NA	NA	NA	NA	NA	NA	NA	26.6	3.2	NA	NA	NA	NA

*Family history of colorectal cancer.

Among the 20 studies, 6558 patients were allocated to control groups, and the sample size of the control groups in the studies ranged from 62 [6] to 1202 [18] patients. The percentage of males ranged from 57.7% [26] to 85.5% [7]. The mean patient age varied from 57 [9, 16] to 66.4 [20].

Data for aspirin users were reported in 9 RCTs [7, 11-14, 16, 18, 20, 22], and the percentage of aspirin users, when reported, ranged from 15.7% [18] to 41% [12]. Twelve RCTs [5-8, 10, 13, 14, 16, 18, 20, 22, 27] reported current smoking status, and the proportion of current smokers varied greatly, from 8.4% [7] to 83.1% [27]. Among the RCTs reporting information on daily calcium use [7, 12, 15, 20, 22-24], the use of daily calcium ranged from 672 mg/d [7] to 1131.2 mg/d [15]. Data regarding daily alcohol use were missing from many studies [5, 6, 8-11, 13, 14, 18, 22-27] and ranged from 8.3 g/d [20] to 12.5 g/d [7] among the studies reporting this factor. Only 4 RCTs [12, 15, 16, 22]] provided data on daily folate use, which varied from 297.6 μg/d [15] to 328 μg/d [22] in the studies reporting this factor. The proportion of family histories of colorectal cancer were reported in 12 studies [6, 7, 10-13, 15, 16, 18, 20, 22, 23] and ranged from 15.3% to 39.4%.

Methodological quality scores varied from 4 [27] to 10 [5-7, 12, 15, 16, 20] on a scale of 2 to 10 (Supplementary Table 2). With respect to the quality of the RCTs, all but two [10, 27] adopted a sufficiently efficacious randomization procedure, and all trials reported an adequate follow-up. Inappropriate blinding was employed in 6 trials [8, 11, 14, 18, 26, 27]. Eighteen RCTs (90%) were scored as high-quality (≥6 points) studies.

### Recurrence rates

**Figure 2:**
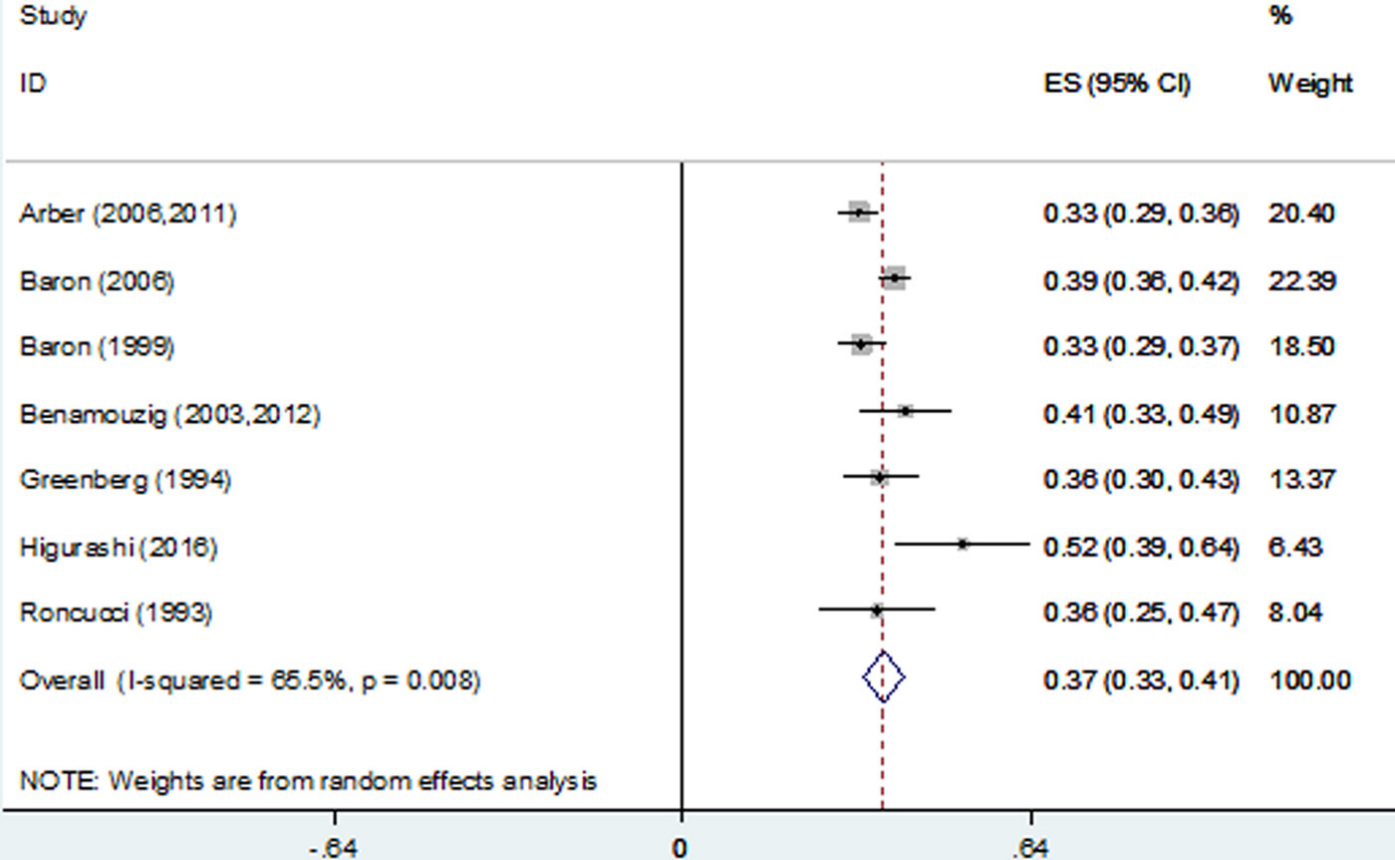
Forest plot of 1-year recurrence rates using a random-effects model

The pooled estimate of the 1-year recurrence rate was 37% (95% confidence interval [CI], 33%-41%; range, 33%-52%), and significant heterogeneity was found among the RCTs, P < 0.001 (Figure 2). The pooled estimate of the 1-year advanced adenoma recurrence rate was 10% (95% CI, 6%-15%; range, 7%-13%), and significant heterogeneity was found among the RCTs, P < 0.001 (Supplementary Figure 1).

The pooled estimate of the 2-year recurrence rate was 47% (95% CI, 41%-54%; range, 46%-51%), and significant heterogeneity was found among the RCTs, P < 0.001 (Supplementary Figure 2).

**Figure 3:**
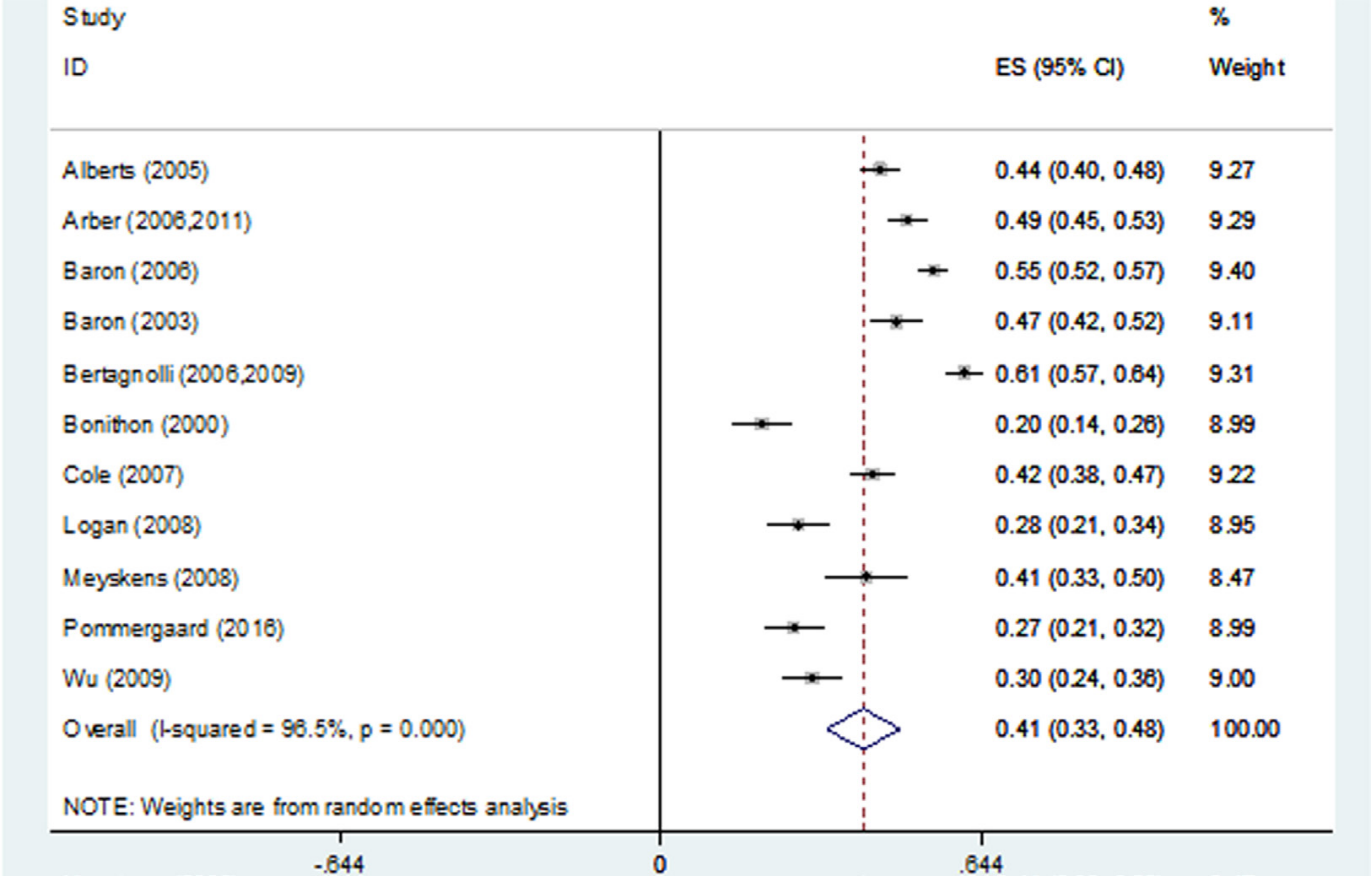
Forest plot of 3-year recurrence rates using a random-effects model

**Figure 4:**
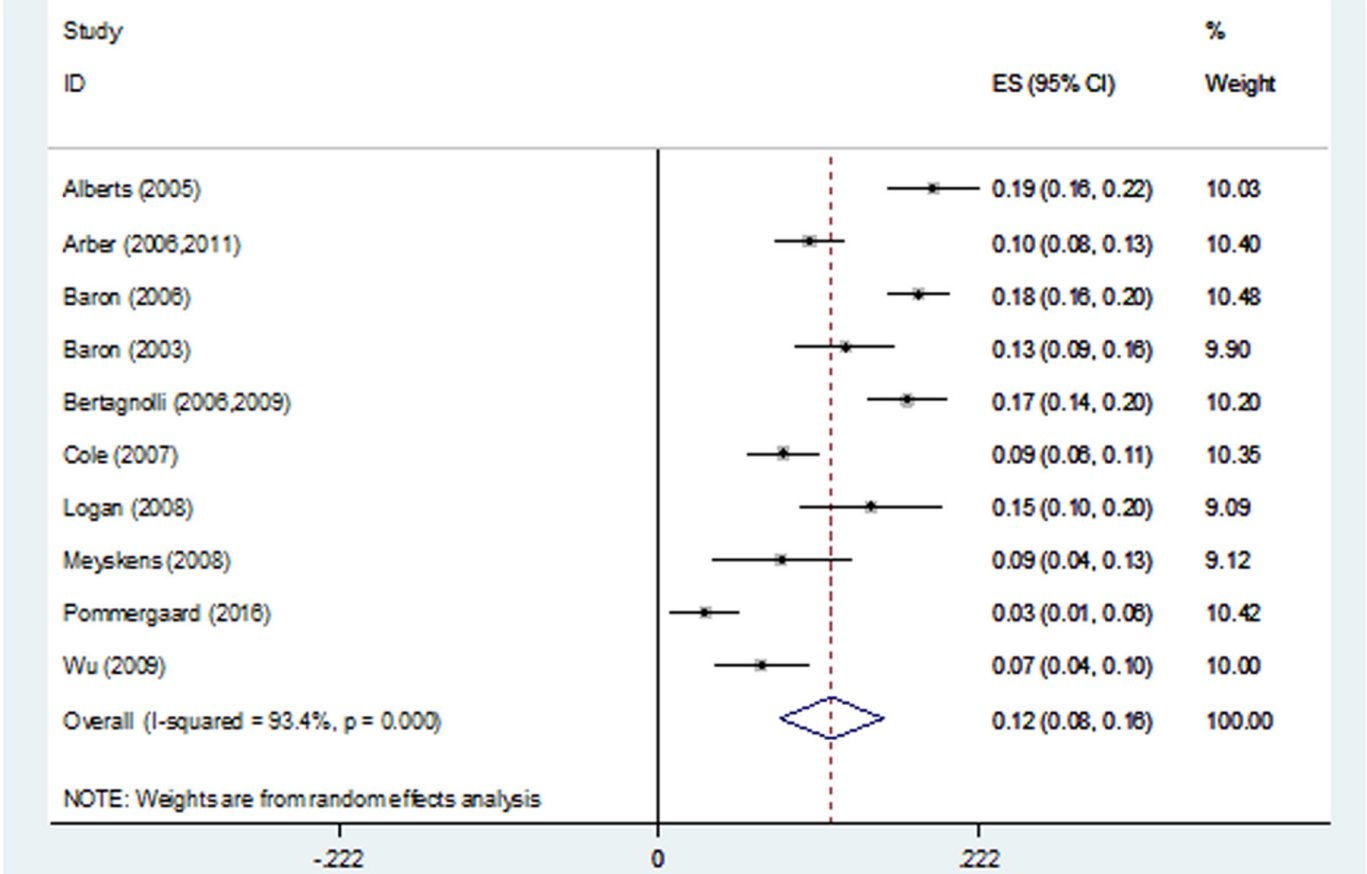
Forest plot of 3-year advanced adenoma recurrence rates using a random-effects model

The pooled estimate of the 3-year recurrence rate was 41% (95% CI, 33%-48%; range, 20%-61%), and significant heterogeneity was found among the RCTs, P < 0.001 (Figure 3). The pooled estimate of the 3-year advanced adenoma recurrence rate was 12% (95% CI, 8%-16%; range, 3%-19%), and significant heterogeneity was found among the RCTs, P < 0.001 (Figure 4).

The pooled estimate of the 4-year recurrence rate was 48% (95% CI, 38%-57%; range, 37%-53%), and significant heterogeneity was found among the RCTs, P < 0.001 (Supplementary Figure 3). The pooled estimate of the 4-year advanced adenoma recurrence rate was 14% (95% CI, 10%-18%; range, 13%-16%), and significant heterogeneity was found among the RCTs, P < 0.001 (Supplementary Figure 4).

The pooled estimate of the 5-year recurrence rate was 60% (95% CI, 52%-68%; range, 48%-68%), and significant heterogeneity was found among the RCTs, P < 0.001 (Supplementary Figure 5). The pooled estimate of the 5-year advanced adenoma recurrence rate was 14% (95% CI, 10%-19%; range, 9%-21%), and significant heterogeneity was found among the RCTs, P < 0.001 (Supplementary Figure 6).

Using univariate regression, none of the 11 variables assessed was associated with an increase in either the 1-year or 3-year adenomas recurrence rate (Supplementary Table 3).

### Subgroup analysis

Subgroup analyses of the 1- and 3-year recurrence rates and the 3-year advanced adenoma recurrence rates were performed to evaluate whether the recurrence was inconsistent depending on the publication year, number of centres, scores of study validity, male gender percentage, mean age, percentage of aspirin users, current smokers, and family histories of colorectal cancer, as well as the use of daily calcium, alcohol, and folic acid (Table 2).

**Table 2:** Stratum-specific recurrence rates according to the studied features

Study Characteristics	1-year recurrence, any			3-year recurrence, any			3-year recurrence, advanced		
	R (95%CI)	I^2^(%)	P_heterogeneity_	R (95%CI)	I^2^(%)	P_heterogeneity_	R (95%CI)	I^2^(%)	P_heterogeneity_
Publication year									
<2000	0.34 (0.31-0.38)	0	0.682	-	-	-	-	-	-
≥2000	0.39 (0.33-0.45)	79	0.003	0.41 (0.33-0.48)	96.5	<0.001	0.12 (0.08-0.16)	93.4	<0.001
Centers									
<10	0.38 (0.31-0.44)	61	0.053	0.44 (0.41-0.46)	0	0.502	0.12 (0.07-0.17)	89.2	<0.001
≥10	0.37 (0.32-0.42)	78.1	0.01	0.39 (0.27-0.50)	97.8	<0.001	0.12 (0.07-0.17)	95.4	<0.001
Study validity									
<6	0.36 (0.25-0.47)	-	-	-	-	-	-	-	-
≥6	0.37 (0.33-0.41)	71.3	0.004	0.41 (0.33-0.48)	96.5	<0.001	0.12 (0.08-0.16)	93.4	<0.001
Male gender(%)									
<70	0.39 (0.36-0.42)	0	0.556	0.40 (0.33-0.48)	96.8	<0.001	0.12 (0.08-0.16)	94	<0.001
≥70	0.37 (0.32-0.41)	65.2	0.022	0.41 (0.33-0.50)	-	-	0.09 (0.04-0.13)	-	-
Mean age(year)									
<60	0.39 (0.37-0.42)	0	0.673	0.42 (0.31-0.54)	97.5	<0.001	0.14 (0.10-0.18)	88.8	<0.001
≥60	0.36 (0.31-0.40)	56.1	0.059	0.38 (0.30-0.47)	92.8	<0.001	0.10 (0.04-0.15)	93.7	<0.001
Aspirin user(%)									
<30	0.36 (0.29-0.43)	87.8	0.004	0.49 (0.43-0.56)	89.3	<0.001	0.16 (0.10-0.21)	92.7	<0.001
≥30	-	-	-	0.44 (0.34-0.55)	95.6	<0.001	0.11 (0.07-0.15)	86.5	<0.001
Current smoker(%)									
<20	-	-	-	0.49 (0.40-0.58)	94.4	<0.001	0.14 (0.09-0.19)	90.8	<0.001
≥20	0.42 (0.36-0.47)	46.3	0.155	0.41 (0.22-0.60)	97.3	<0.001	0.10 (-0.01-0.20)	97.6	<0.001
Daily calcium(mg/d)									
<1000	0.33 (0.29-0.37)	-	-	0.41 (0.32-0.50)	90	<0.001	0.13 (0.06-0.20)	92.3	<0.001
≥1000	-	-	-	0.24 (0.17-0.31)	64.7	0.093	0.15 (0.10-0.20)	-	-
Daily alcohol(g/d)									
<10	-	-	-	0.39 (0.32-0.47)	86.8	0.001	0.12 (0.05-0.19)	94	<0.001
≥10	-	-	-	0.28 (0.21-0.34)	-	-	0.15 (0.10-0.20)	-	-
Daily folate(ug/d)									
<300	-	-	-	0.28 (0.21-0.34)	-	-	0.15 (0.10-0.20)	-	-
≥300	-	-	-	0.40 (0.31-0.49)	89.3	<0.001	0.09 (0.06-0.13)	67.9	0.044
*Family history (%)									
<25	0.39 (0.32-0.46)	85.2	0.001	0.46 (0.33-0.59)	97.8	<0.001	0.15 (0.10-0.20)	91.6	<0.001
≥25	0.41 (0.33-0.49)	-	-	0.38 (0.32-0.45)	89.7	<0.001	0.12 (0.08-0.17)	88.7	<0.001

*Family history of colorectal cancer.

Heterogeneity of the 1-year recurrence rate was less significant among studies published before 2000, those in which the number of centres was < 10, those in which the male gender percentage was < 70%, those in which the mean age was < 60 or ≥ 60 years, or those in which the percentage of smokers was ≥ 20%.

Heterogeneity of the 3-year recurrence rate was less significant among studies in which the number of centres was < 10 and those in which the daily calcium intake was ≥ 1000 mg/d. Heterogeneity persisted in 4 strata: mean age, aspirin use, current smoker, and a family history of colorectal cancer.

Heterogeneity of the 3-year advanced adenoma recurrence rate was significant among all studies.

The sensitivity analysis excluded two RCTs [11, 13] that did not exclude patients with familial adenomatous polyposis syndrome; the results showed that the pooled estimate of the 1-year recurrence rate was 38% (95% CI, 34-42), with no significant heterogeneity (P = 0.052).

### Publication bias

The Begg funnel plots for 1-year recurrence rate and 1-year advanced adenoma recurrence rate are shown in Supplementary Figures 7 and 8. These plots and Egger’s test of 1-year recurrence rate and 1-year advanced adenoma recurrence rate for publication bias showed that the risk of having missed or overlooked trials was insignificant: P values of 0.861 and 0.64, respectively, were obtained using Egger’s test.

The Begg funnel plots for 3-year recurrence rate and 3-year advanced adenoma recurrence rate are shown in Supplementary Figures 9 and 10. These plots and Egger’s tests of 3-year recurrence rate and 3-year advanced adenoma recurrence rate for publication bias showed that the risk of having missed or overlooked trials was significant: P values of <0.001 and 0.003, respectively, were obtained using Egger’s test.

The Begg funnel plots for the 4-year recurrence rate are shown in Supplementary Figure 11. This plot and Egger’s tests of the 4-year recurrence rate for publication bias showed that the risk of having missed or overlooked trials was insignificant: a P value of 0.505 was obtained using Egger’s test.

The Begg funnel plots for the 5-year recurrence rate and 5-year advanced adenoma recurrence rate are shown in Supplementary Figures 12 and 13. This plot and Egger’s tests for publication bias for 5-year recurrence rates showed that the risk of having missed or overlooked trials was insignificant: a P value of 0.067 was obtained using Egger’s test. The plot and Egger’s test for publication bias for the 5-year advanced adenoma recurrence rate showed that the risk of having missed or overlooked trials was significant: a P value of 0.04 was obtained using Egger’s test.

## DISCUSSION

The recurrence rate of colorectal adenoma in control groups among RCTs may be a reliable measure of the spontaneous course of the disease and a basic measure for calculating sample size in clinical trials evaluating new drugs for the prevention of colorectal adenoma. The homogeneity of adenoma recurrence or advanced adenoma recurrence rates is a conducive condition for acquiring dependable data regarding the natural history of the disease and for calculating sample size. The heterogeneity of adenoma recurrence and advanced adenoma recurrence rates among studies probably illustrates variability in the patient selection and in the evaluation of adenoma recurrence between observers.

This meta-analysis of the colorectal adenoma recurrence rates of placebo-controlled groups shows that heterogeneity of the adenoma recurrence or advanced adenoma recurrence rates of post-polypectomy patients with colorectal adenoma who received placebo in RCTs is a general characteristic of these studies. Differences between the RCTs were apparent: adenoma recurrence rates ranged from 33% to 56% at 1 year, from 46% to 51% at 2 years, from 20% to 61% at 3 years, from 37% to 52% at 4 years, and from 48% to 68% at 5 years; advanced adenoma recurrence rates ranged from 7% to 13% at 1 year, from 3% to 19% at 3 years, from 13% to 16% at 4 years, and from 9% to 21% at 5 years. In our analysis, the pooled adenoma recurrence rate estimated using the random-effects model was 37% at 1 year, 47% at 2 years, 41% at 3 years, 48% at 4 years, and 60% at 5 years, and the pooled advanced adenoma recurrence rate was 10% at 1 year, 12% at 3 years, 14% at 4 years, and 14% at 5 years. Although the numbers of included patients in the studies at 1 and 3 years were large, indicating that the estimated adenoma recurrence and advanced adenoma recurrence rates were robust, the confidence intervals of the estimated adenoma recurrence rates at 1 year (95% CI, 33%-41%) and at 3 years (95% CI 33%-48%) and the estimated advanced adenoma recurrence rate at 3 years (95% CI, 8%-16%) remained wide. This inconsistency among studies is not surprising considering potential systematic biases in the selection of patients with various demographic and clinical features, the different timing of outcome evaluation, measurement bias of outcome among the observers, and differences in the histopathological features of adenomas resected before inclusion.

Due to a lack of data regarding the 2-year, 4-year, and 5-year adenoma recurrence rates and regarding the 1-year, 2-year, 4-year, and 5-year advanced adenoma recurrence rates, we analysed the 1- and 3-year adenoma recurrence rates and the 3-year advanced adenoma recurrence rate in an attempt to explain the significant inconsistency regarding the natural course of placebo-controlled colorectal adenoma by stratifying the studies according to variables that reflected the subjects studied and the study design features.

Heterogeneity in recurrence remained among the studies after stratifying the subjects and study characteristics, and apparent heterogeneities in adenoma recurrence at 1 year or at 3 years and in advanced adenoma recurrence at 3 years remained even in the stratum of high-quality studies, suggesting that the heterogeneity was not explained solely by study validity. Although heterogeneity in the adenoma recurrence rate at 1 year was less significant in the stratum of studies including a high percentage of current smokers, we observed that the heterogeneity in the adenoma and advanced adenoma recurrence rates at 3 years remained after stratifying the patients and studies according to the proportions of current smokers. Among the studies, the study by Meyskens [14] had the highest proportion of current smokers (41.4%), and that of Alberts [20] had the lowest proportion of current smokers (43.9%); the recurrence rates of these studies were 41.1% and 43.9%, respectively. This finding indicates that the heterogeneity was not explained by the proportions of current smokers. In the studies by Pommergaard [5], it was suggested that aspirin, calcium and calcitriol might be harmful for current smokers in terms of recurrence, and Ishikawa [8] also considered that the use of aspirin in smokers might increase the risk of recurrence. Consistent with the former studies, we might infer that smoking did not influence recurrence alone but did when combined with aspirin use. We also observed that heterogeneities in the adenoma recurrence rate at 1 year and in the adenoma or advanced adenoma recurrence rate at 3 years remained after stratifying the patients and studies according to the proportion of aspirin users. Given the results of earlier meta-analyses [28, 29], the lack of an observed effect of aspirin in our analysis was surprising. We then searched for and reviewed guidelines for the management of acute coronary syndromes and found that current guidelines recommend the indefinite use of 75 to 162 mg of aspirin in all patients with documented coronary artery disease [30–32]. However, an earlier study [5] found that an aspirin dose of 75 mg did not affect adenoma recurrence. Thus, a lack of data in the studies regarding the daily aspirin dose taken by the patients might have affected the accuracy of the results.

Heterogeneity in adenoma recurrence at 1 year remained in the studies that were published after 2000, in studies involving numerous centres, and in studies involving high proportions of males. Heterogeneity was less significant in RCTs published before 2000 and in RCTs including few centres, and we found that the studies published before 2000 were conducted in fewer centres, suggesting that the heterogeneity might have been lower had the patients been selected for greater consistency in demographic characteristics. Homogeneity in adenoma recurrence at 1 year was seen in the studies with a proportion of males < 70% and in studies that included patients whose mean age was < 60 or ≥ 60 years. However, no homogeneity in adenoma recurrence or advanced adenoma recurrence was observed at 3 years, confounding these findings.

Using a univariate analysis, no variable was associated with both adenoma and advanced adenoma recurrence.

This analysis was based on summary data, and more detailed comparisons of recurrence might be made by performing a meta-analysis of individual patient data. Concomitantly, we realize that it may be impossible to collect individual patient data from every study, indicating that the studies from which we acquired the data may represent a biased sample of the available studies.

Finally, we must be cognizant of publication bias in settings in which many small studies are being conducted. Publication bias may occur; thus, studies indicating an apparent reduction of recurrence tend to be published more often than those indicating no distinction. The risk of having missed or overlooked RCTs in the setting of studies evaluating recurrence in patients with post-polypectomy colorectal adenoma was substantial. Therefore, it is possible that small studies with a low rate of recurrence or with a small drug effect remained preferentially unpublished. However, the adenoma or advanced adenoma recurrence rate at 1 year, and the adenoma recurrence rate at 4 or 5 years probably reflect no substantial publication bias, and such considerations are believed unlikely to change the magnitude of our pooled estimate rates.

In conclusion, the adenoma recurrence rates among post-polypectomy colorectal adenoma patients in the placebo-controlled groups were variable, and no single design variable was identified that explained the heterogeneity among the placebo arm outcomes for recurrence.

This meta-analysis indicates that conducting an RCT that measures a long-term endpoint is problematic in terms of endoscopic adenoma recurrence due to the high variability among the placebo arm rates. Moreover, we should not compare various RCTs with different follow-up durations or the use of different drugs because the differences in recurrence rates may be related to the variations in the demographic and baseline characteristics among the included subjects.

## MATERIALS AND METHODS

### Selection of randomized trials

This analysis was performed in accordance with the PRISMA (Preferred Reporting Items for Systematic Reviews and Meta-Analyses) statement [33]. The primary sources of the reviewed studies, exclusively in English, were PubMed, the Cochrane Controlled Trials Register, and Web of Science; the following medical subject headings were used: *adenoma or adenomatous polyp or polyps*, *colorectal or colon or rectum or large bowel, recurrence or relapse*, and *randomized or randomized trial or clinical trial*. The search included literature published through July 2016. The computer search was supplemented with manual searches of the reference lists of all available review articles, primary studies, meeting abstracts, and bibliographies of books to identify other studies not identified in the computer search. When the results of a single study were reported in more than one publication, only the most recent and complete data were included in the meta-analysis.

Studies were included in the analysis when (1) they were RCTs comparing any therapy with placebo or no treatment in colorectal adenoma; (2) colorectal adenoma status was assessed using complete colonoscopy and no adenomas were knowingly left in the large bowel at enrolment; (3) adenoma recurrence was assessed as an outcome measure of the effect of treatment by colonoscopic follow-up; and (4) they were published or accepted for publication as full-length articles or abstracts. Decisions on which RCTs to include were made by two reviewers (X.S. and Z.Y.) who were not blinded. Queries concerning inclusion were resolved by discussion and consensus between these two reviewers. Excluded studies were identified together with the reason for exclusion. Among the 1545 articles reviewed, 20 RCTs [5-16, 18, 20, 22-27] met the inclusion criteria. Studies were excluded when the recurrence rates were not reported [34, 35]; they compared recurrence rates regarding colorectal cancer (CRC) [36]; or no definite follow-up duration was provided [37].

### Review of the trials

The trials were first reviewed using a list of predefined, pertinent issues relating to patient characteristics and treatments. One reviewer (X.S.) extracted data from the included trials into predesigned forms and assessed the quality of the trials based on the quality criteria suggested by Jadad [38] and Banares [39] (Supplementary Table 4). Data extraction and quality assessment were then evaluated thoroughly by another reviewer (Z.Y.). The aim of the quality assessment was to evaluate factors relating to the quality of the reported allocation, randomization and blinding processes, the comparability of the treatment and control groups, and the suitability and quality of the analyses performed. The quality of the trials was evaluated based on each separate component. The maximum possible score was 10 points.

### Statistical analyses

Pooled estimates of the placebo-controlled groups’ endoscopic recurrence rates were calculated using a random-effects logistic regression analysis after applying sample weights based on the placebo sample size as implemented using Stata software (version 12.0; Stata Corporation, College Station, TX, USA). The assumption of heterogeneity implied by the utilization of random-effect models was justified by the differences in the patients’ features and study characteristics. Three methods were used to explore and explain the diversity among the studies: (1) stratum analysis of variables suspected of causing inconsistency; (2) meta-regression; and (3) subgroup analysis. Therefore, stratum-specific rates of endoscopic recurrence for different patient-level and study-level covariates were calculated. We used 11 stratifying variables: publication year, number of participating centres, study location, mean age, proportion of males, proportion of aspirin users, proportion of current smokers, proportion of family histories of CRC, use of calcium, use of alcohol, and use of folic acid. Only univariate regression models were used to examine the association between study design and the recurrence rates in placebo-controlled groups. We did not consider using multivariate analysis because of the wide heterogeneity and lack of complete data to identify possible variables that might explain heterogeneity. The correlation between continuous measures of the study characteristics was assessed using the Pearson correlation coefficient.

Begg’s funnel plots were generated, and Egger’s regression asymmetry test was used to examine potential publication bias [40]. For all analyses, P < 0.05 was considered to indicate statistical significance. All analyses were performed using Stata (version 12.0; Stata Corporation, College Station, TX, USA).

## SUPPLEMENTARY MATERIALS FIGURES AND TABLES



## Abbreviations

CRA: colorectal adenoma
CRC: colorectal cancer
RCT: randomized controlled trial
CI: confidence interval
