# Supplementary material for: Colorectal adenoma recurrence rates among post-polypectomy patients in the placebo-controlled groups of randomized clinical trials: a meta-analysis
Source: Oncotarget. 2017 May 25;8(37):62371–81. doi: 10.18632/oncotarget.18181 (PMC5617512; doi:10.18632/oncotarget.18181)
Supplement: Supplementary file 1 [file oncotarget-08-62371-s001.pdf]

Colorectal adenoma recurrence rates among post-polypectomy patients in the placebo-controlled groups of randomized clinical trials: a meta-analysis

SUPPLEMENTARY MATERIALS

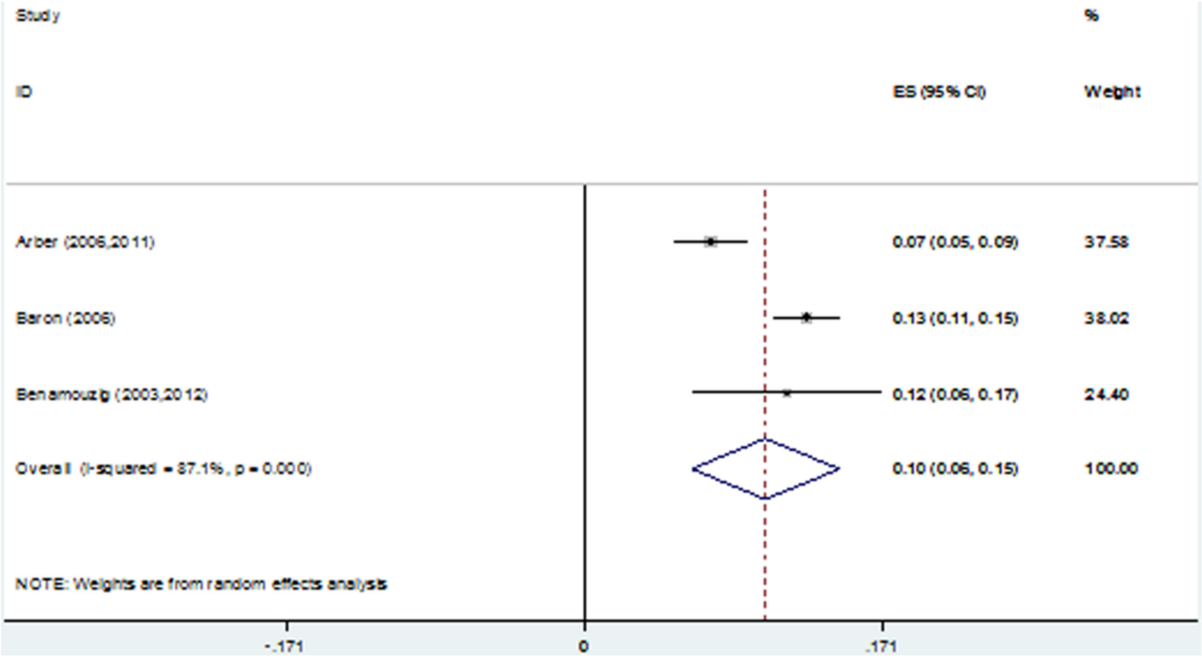

Supplementary Figure 1: Forest plot of 1-year advanced recurrence rates using a random-effects model.

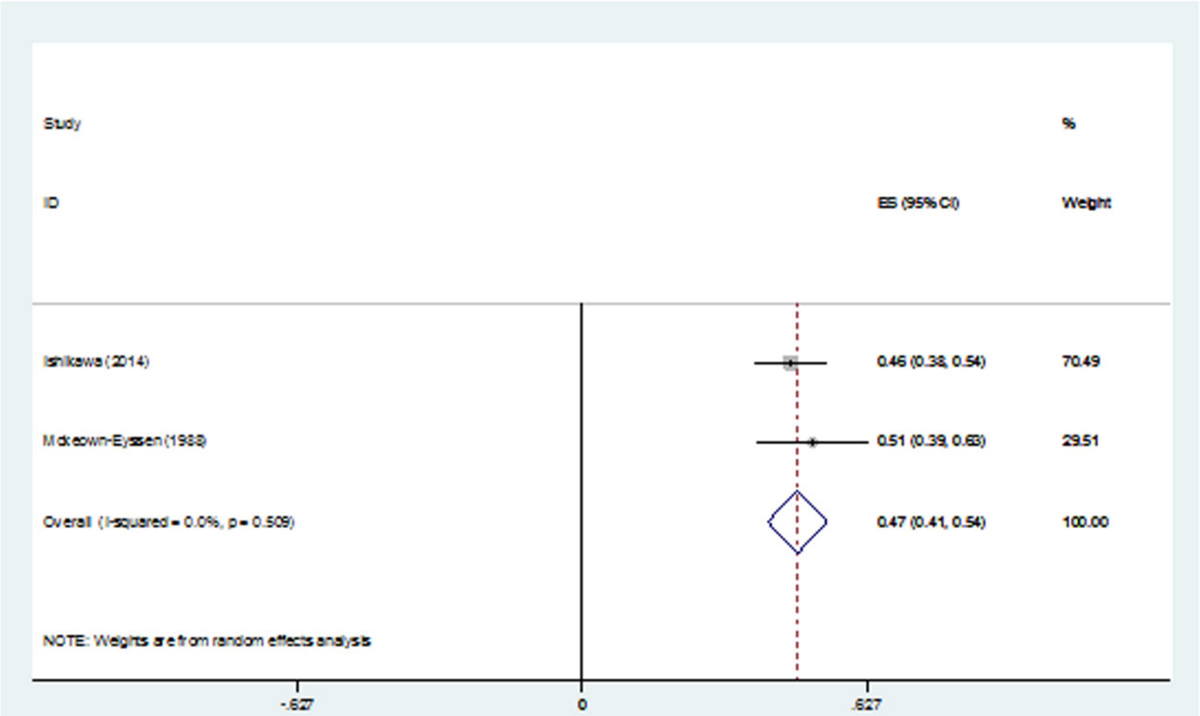

Supplementary Figure 2: Forest plot of 2-year recurrence rates using a random-effects model.

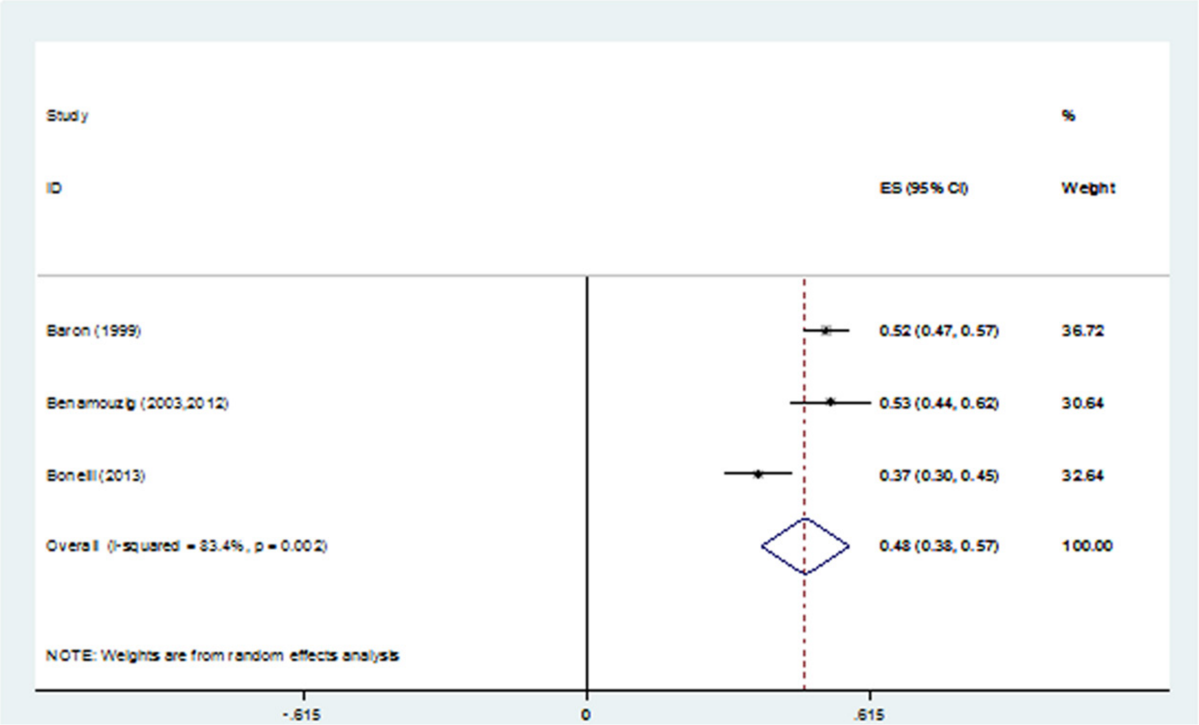

Supplementary Figure 3: Forest plot of 4-year recurrence rates using a random-effects model.

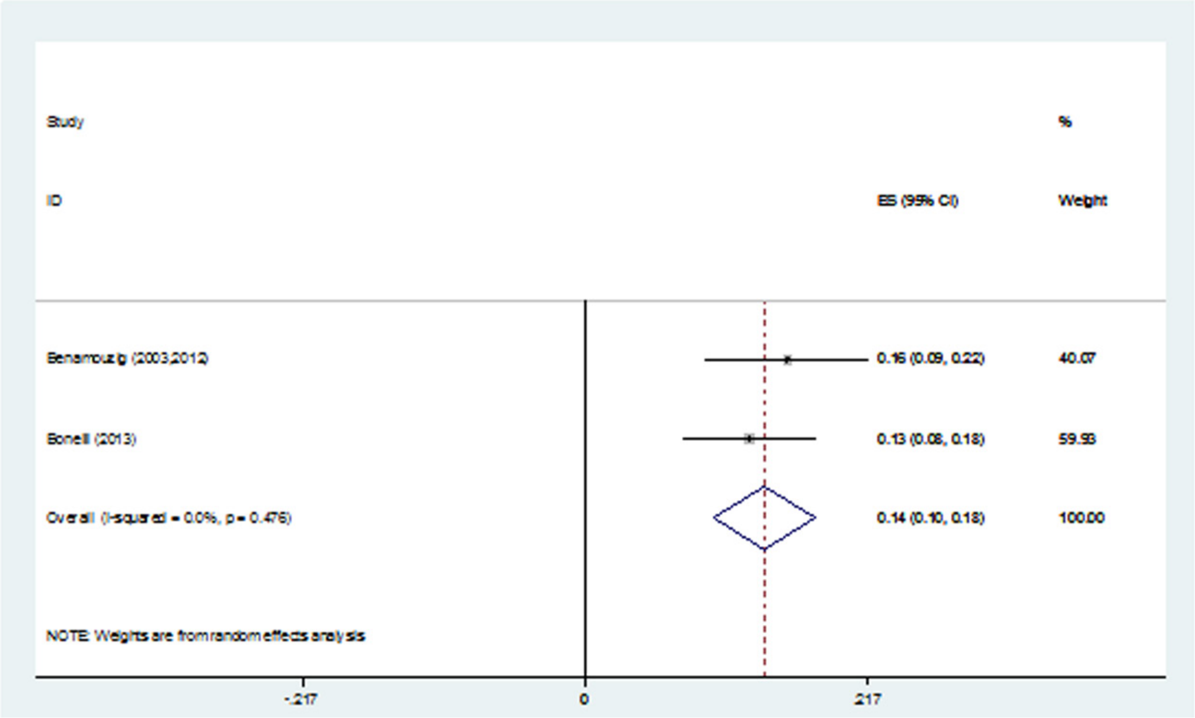

Supplementary Figure 4: Forest plot of 4-year advanced adenoma recurrence rates using a random-effects model.

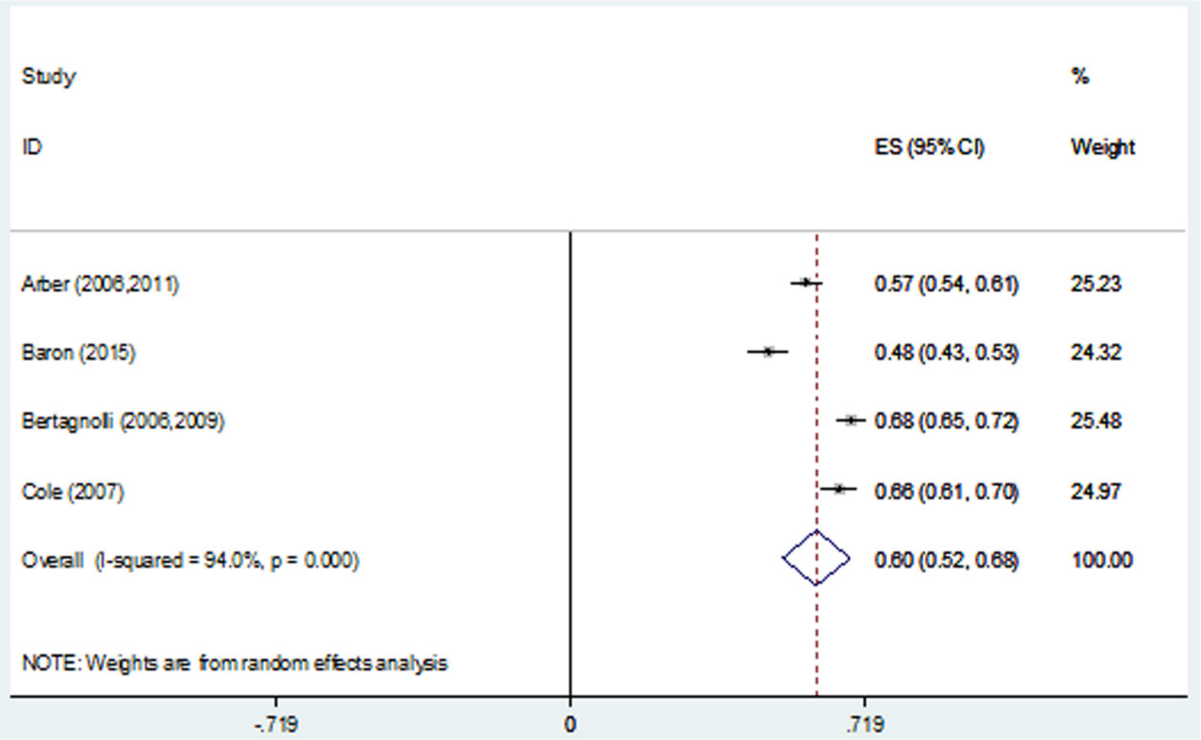

Supplementary Figure 5: Forest plot of 5-year recurrence rates using a random-effects model.

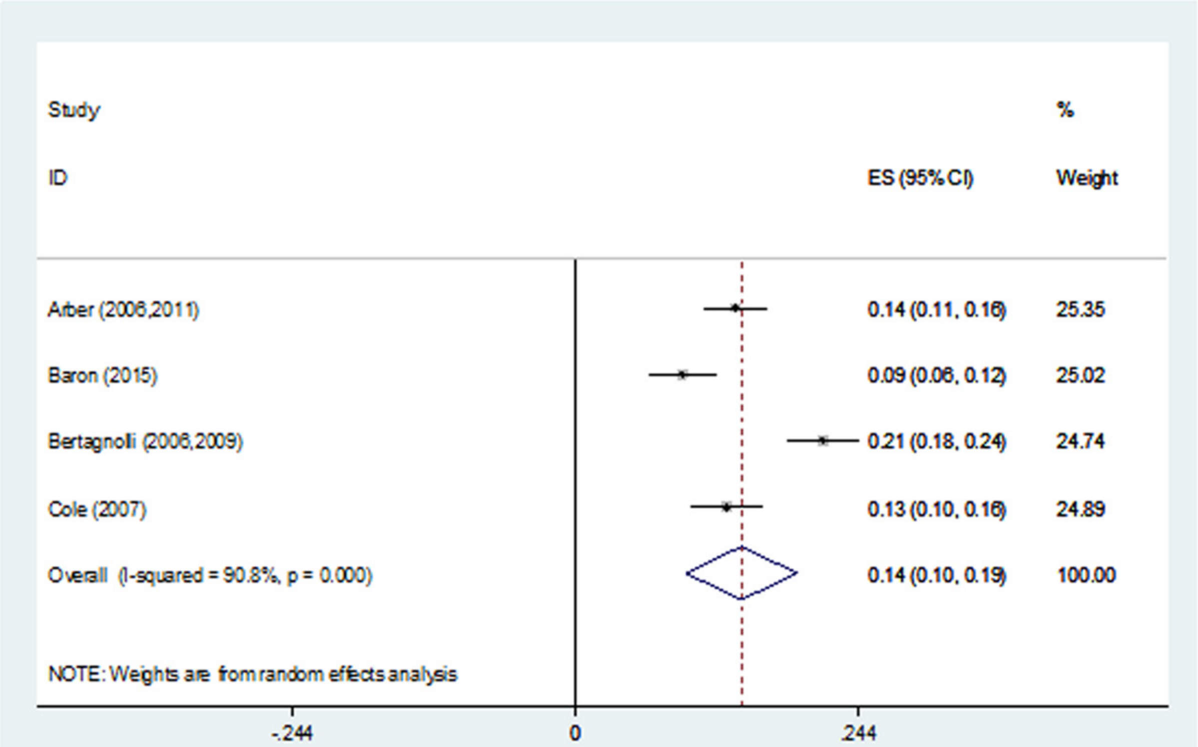

Supplementary Figure 6: Forest plot of 5-year advanced adenoma recurrence rates using a random-effects model.

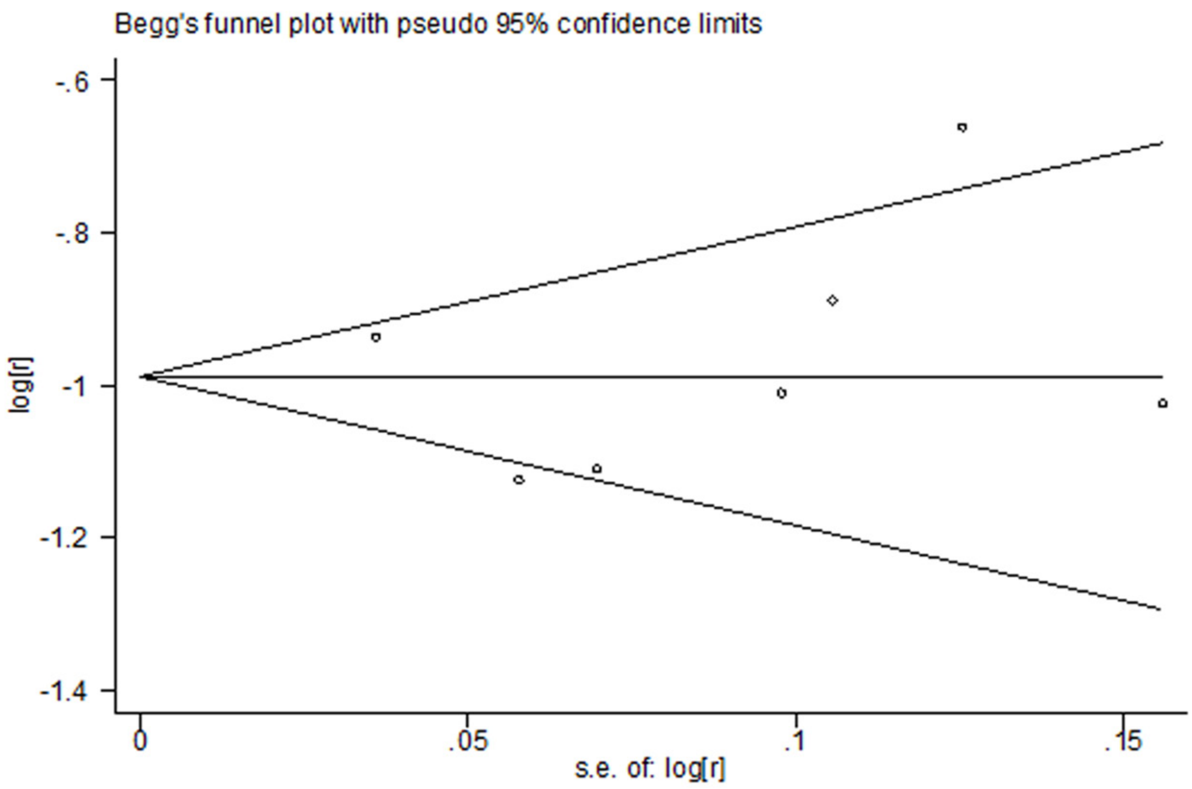

Supplementary Figure 7: Begg's funnel plots for 1-year recurrence rate.

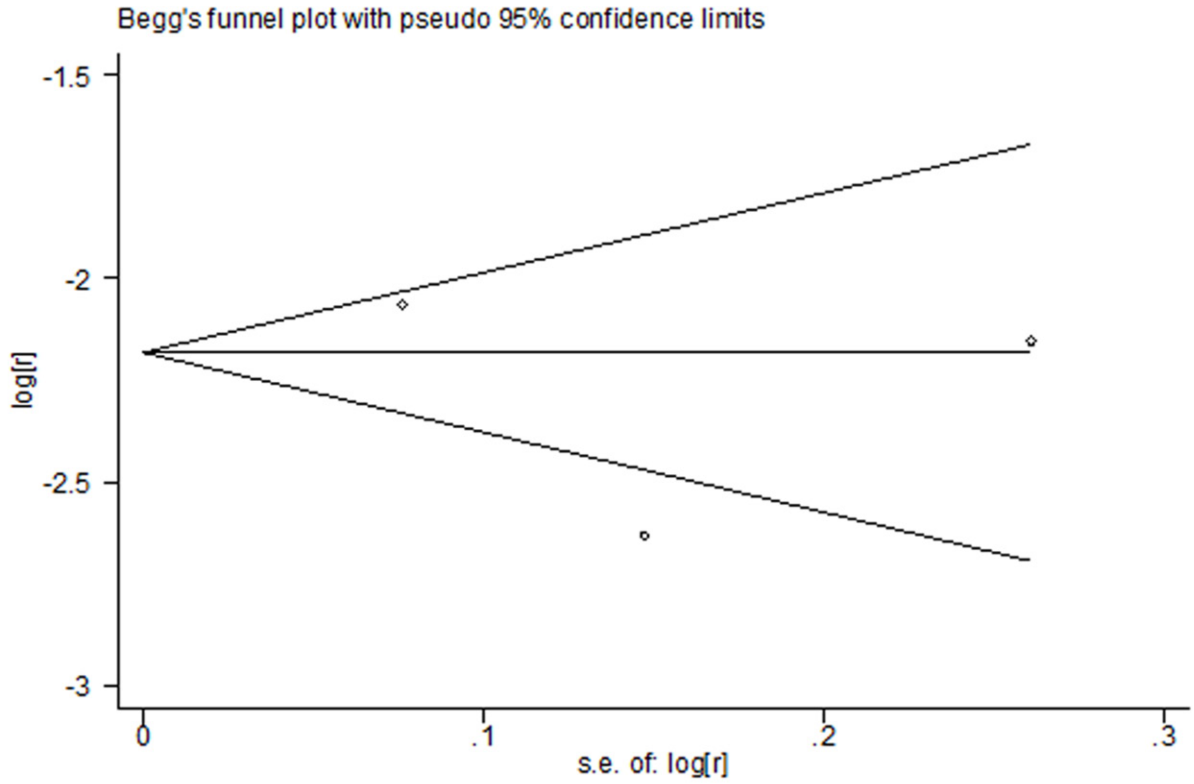

Supplementary Figure 8: Begg's funnel plots for 1-year advanced adenoma recurrence rate.

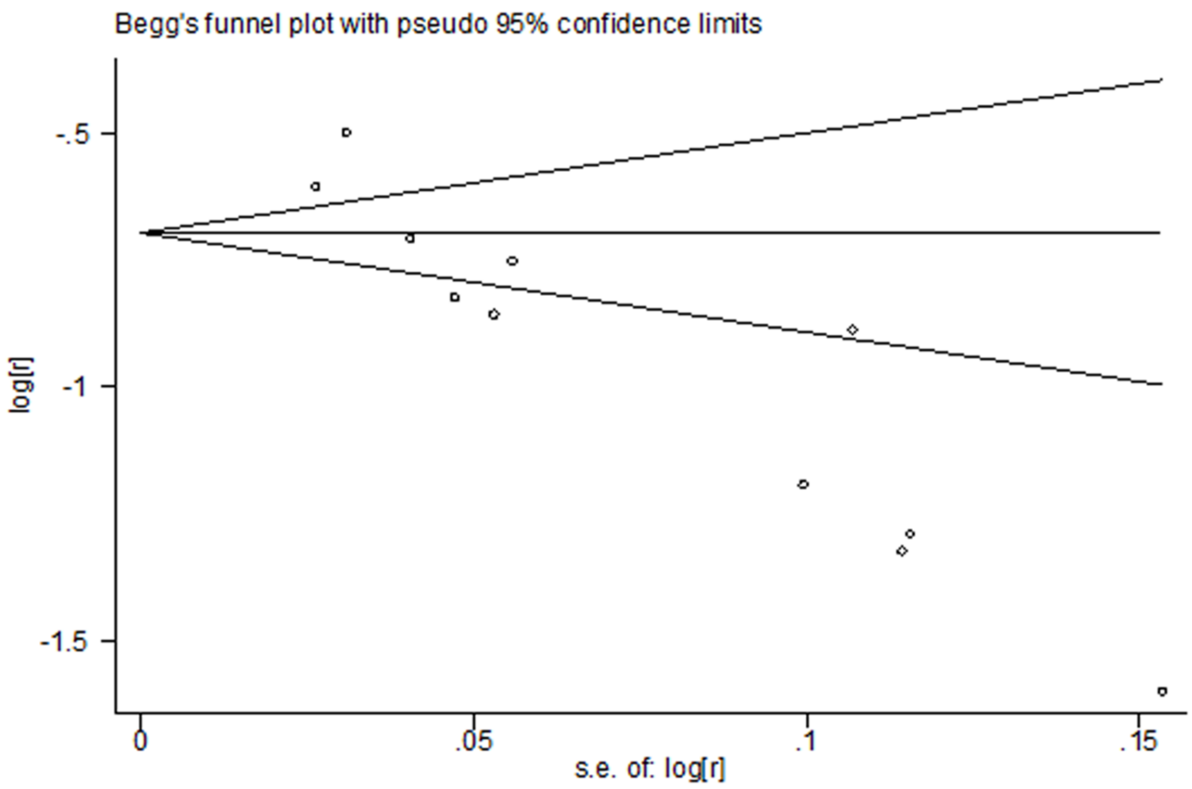

Supplementary Figure 9: Begg’s funnel plots for 3-year recurrence rate.

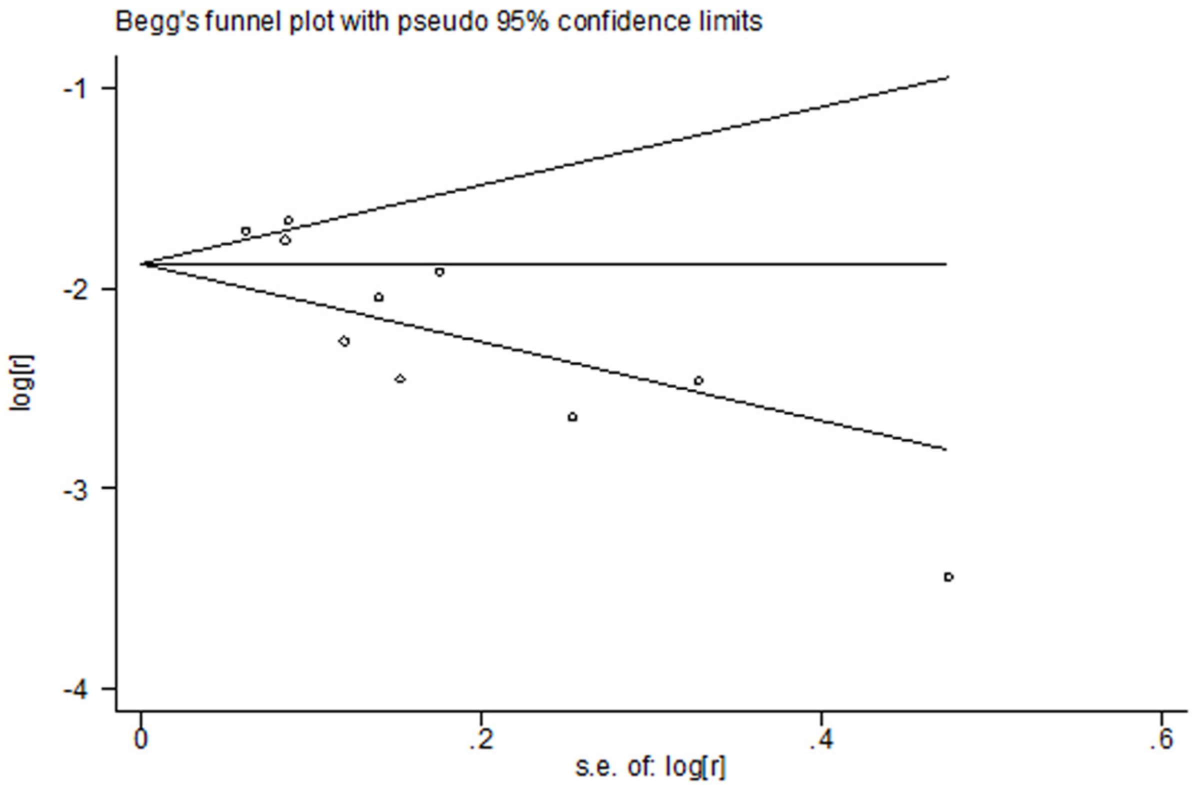

Supplementary Figure 10: Begg's funnel plots for 3-year advanced adenoma recurrence rate.

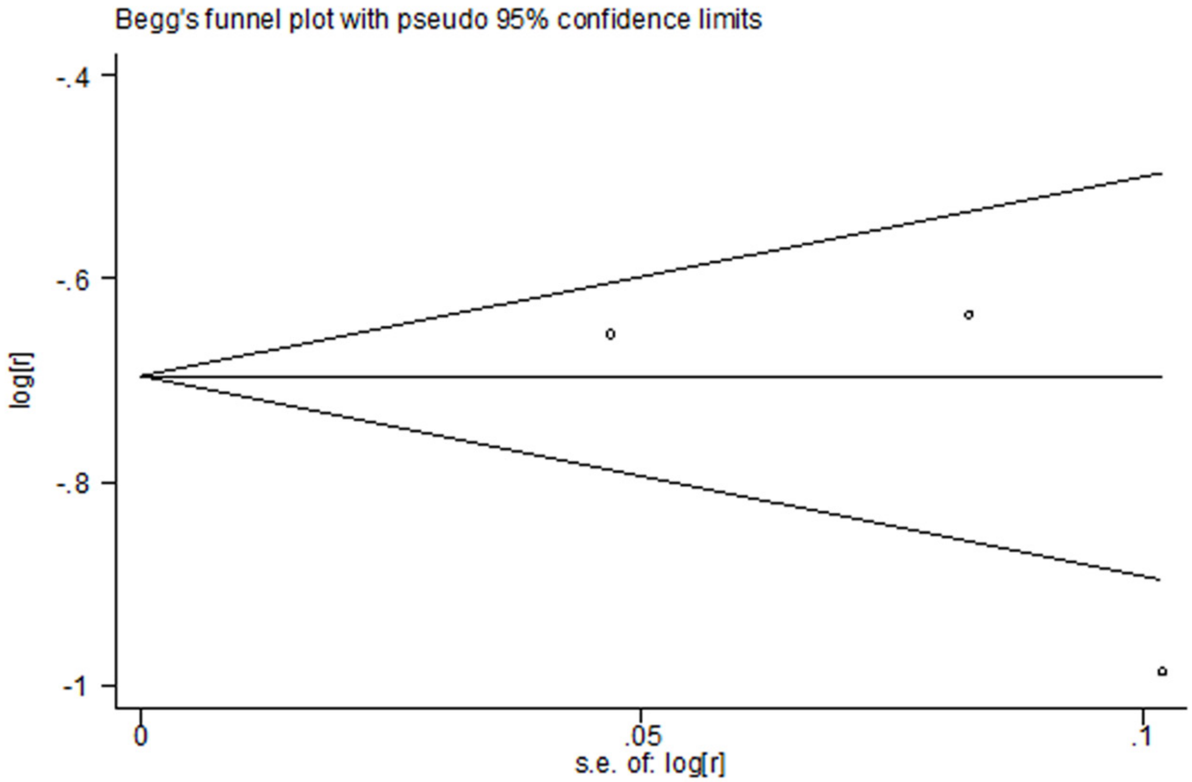

Supplementary Figure 11: Begg's funnel plots for 4-year recurrence rate.

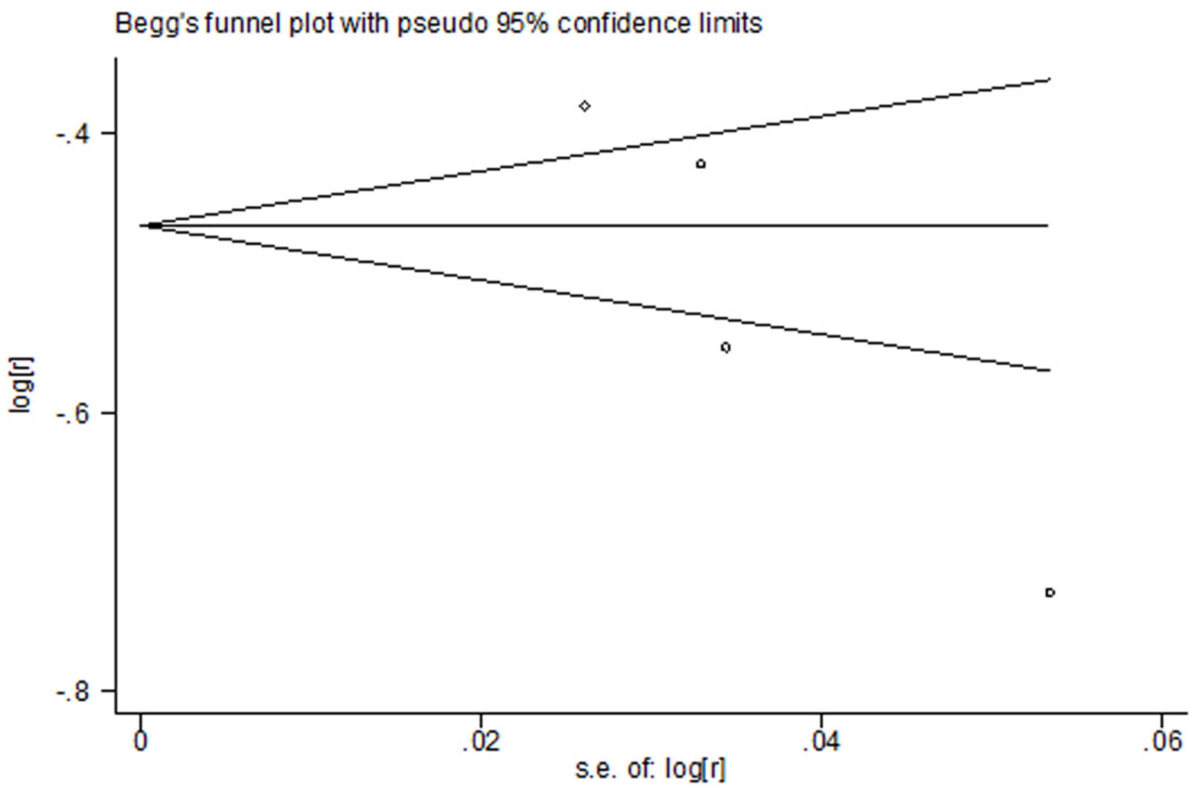

Supplementary Figure 12: Begg's funnel plots for 5-year recurrence rate.

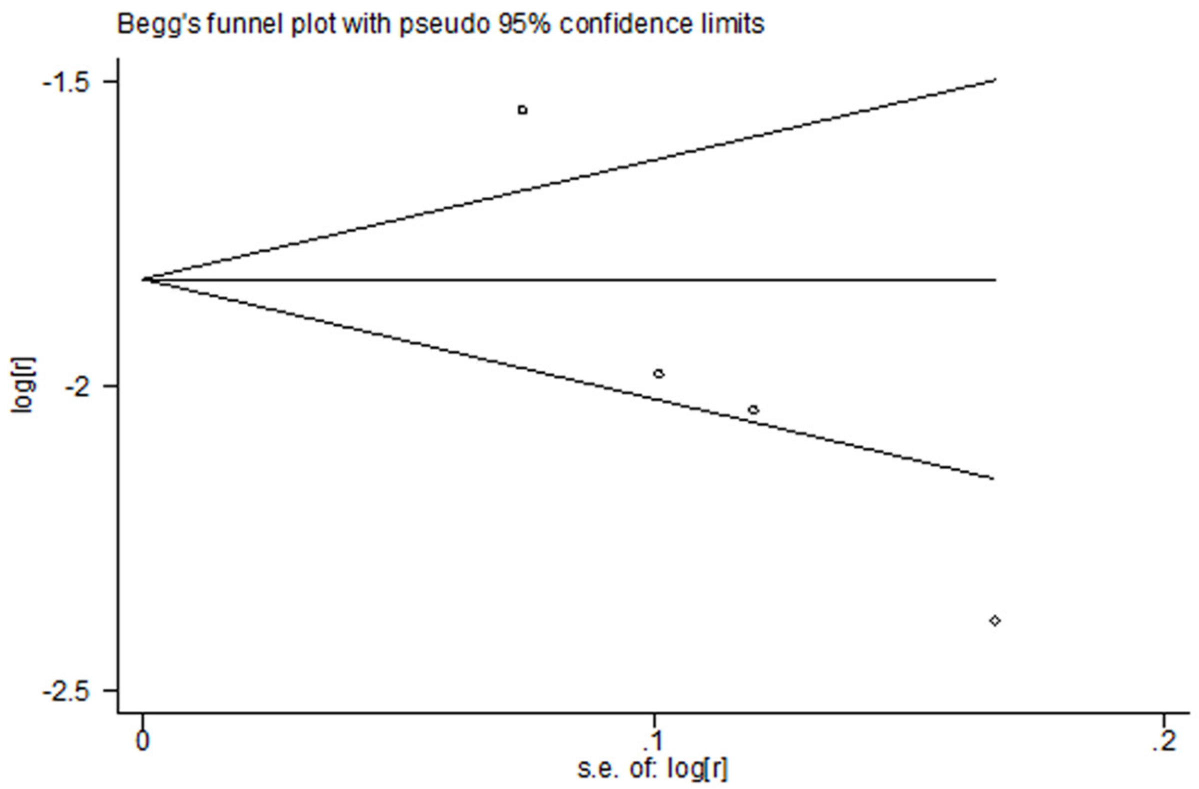

Supplementary Figure 13: Begg's funnel plots for 5-year advanced adenoma recurrence rate.

Supplementary Table 1: Characteristics of treatment and control arms of RCTs included in the meta-analysis

Author	Year	Control arm	Comparison drug: dose and routes of administration
Mckeown	1988	Placebo	Vit C 400mg/d plus Vit E 400mg/d per os
Roncucci	1993	No treatment	Vitamin A 30000IU, vitamin C 1g, plus vitamin E 70mg once daily, or lactulose 20g once or twice daily per os
Greenberg	1994	Placebo	Beta cartone 25mg once daily, or Vit C 1000mg+ Vit E 400mg once daily, or beta cartone 25mg+Vit C 1000mg+ Vit E 400mg once daily per os
Baron	1999	Placebo	calcium 1200mg/d per os
Bonithon	2000	Placebo	Calcium 2000mg/d, or ispaghula husk 3500mg/d per os
Baron	2003	Placebo	aspirin 81mg/d, or aspirin 325mg/d per os
Alberts	2005	Placebo	Ursodeoxycholic Acid(UDCA) 8-10mg/kg of body weight once daily per os
Baron	2006	Placebo	rofecoxib 25mg/d per os
Cole	2007	Placebo	folic acid 1mg/d per os
Logan	2008	Placebo	aspirin 300mg/d, or folate 0.5mg/d, or aspirin 300mg/d plus folate 0.5mg/d per os
Meyskens	2008	Placebo	difluoromethylornithine (DFMO) 500 mg plus sulindac 150 mg once daily per os
Bertagnolli	2009	Placebo	celecoxib 200mg twice daily, or celecoxib 400mg twice daily per os
Wu-Am	2009	Placebo	folic acid 1mg/d per os
Arber	2012	Placebo	celecoxib 400mg/d per os
Benamouzig	2013	Placebo	lysine acetylsalicylate 160mg/d, or lysine acetylsalicylate 300mg/d per os
Bonelli	2014	Placebo	selenium 200mg, zinc 30mg, Vit E 30mg, Vit A 2mg, Vit C 180mg once daily per os
Ishikawa	2014	Placebo	aspirin 100mg/d per os
Baron	2015	Placebo	Vit D 1000IU/d, or Ca 1200mg/d per os
Higurashi	2016	Placebo	metformin 250mg/d per os
Pommergaard	2016	Placebo	calcitriol 0.5mg, acetylsalicylic acid 75mg, plus calcium carbonate 1250 mg once daily per os

Supplementary Table 2: Methodological characteristics of RCTs included in the meta-analysis

Author	Year	Methodological quality					
		Allocation generation	Allocation concealment	Double blinded	Adequate follow-up	Efficacy of randomization	Quality score*
Mckeown	1988	1	1	1	1	0	4
Roncucci	1993	1	1	0	1	2	5
Greenberg	1994	1	1	2	1	2	7
Baron	1999	2	1	2	1	2	8
Bonithon	2000	1	3	2	1	2	9
Baron	2003	2	1	2	1	2	8
Alberts	2005	2	3	2	1	2	10
Baron	2006	2	1	1	1	2	7
Cole	2007	2	3	2	1	2	10
Logan	2008	2	3	2	1	2	10
Meyskens	2008	1	1	1	1	2	6
Bertagnolli	2009	1	1	2	1	2	7
Wu	2009	2	3	2	1	2	10
Arber	2011	2	1	1	1	2	7
Benamouzig	2012	2	3	2	1	0	8
Bonelli	2013	1	2	2	1	2	8
Ishikawa	2014	2	3	1	1	2	9
Baron	2015	2	3	2	1	2	10
Higurashi	2016	2	3	2	1	2	10
Pommergaard	2016	2	3	2	1	2	10

*≥6, high-quality trials.

Supplementary Table 3: Predictors of recurrence among all studies

Study characteristics	1-year recurrence, any			3-year recurrence, any		
	β	SE	P	β	SE	P
Publication year	6	4.764	0.263	-	-	-
Centers	-1.625	5.419	0.776	-5.168	8.235	0.546
Study validity	3.067	7.61	0.704	-	-	-
Male gender(%)	0.325	0.298	0.325	0.634	0.412	0.158
Mean age(year)	1.259	1.553	0.454	-0.394	1.368	0.78
Aspirin user(%)	-	-	-	-0.572	0.31	0.115
Current smoker(%)	0.659	0.054	0.052	-0.305	0.46	0.537
Daily calcium(mg/d)	-	-	-	-0.067	0.034	0.138
Daily alcohol(g/d)	-	-	-	-3.814	1.493	0.125
Daily folate(ug/d)	-	-	-	0.576	0.264	0.161
Family history of colorectal cancer(%)	-0.064	0.516	0.912	-0.06	0.689	0.933

Supplementary Table 4: Characteristics of the treatment and control arms of the RCTs included in the meta-analysis

Allocation sequence generation
Computer-generated of random numbers or similar: 2 points
Not described or inadequate:1
Allocation concealment
Central randomization: 3 points
Sealed envelopes or similar: 2
Not described or inadequate: 1
Double blinding
Identical placebo tablets or double dummy: 2 points;
Double-blind, but method not described: 1;
No double-blinding or inadequate method: 0.
Description of protocol deviations
Numbers and reasons described: 1
Not described: 0.
Efficacy of randomization
Prognostic variables balanced, presented in tabular form: 2 points
Prognostic variables balanced and described in text: 1
No information reported or variables unbalanced: 0.

TOTAL SCORE: 10 points†.
* Modified from Jadad et al. and Bañares et al.
† The ten-point score is ranked high (≥ 6 points) or low (≤ 5 points) to define high and low levels of quality, respectively. For those RCTs for which double-blinding is unfeasible, the maximum score is 8 points.
